# ^19^F NMR Monitoring of Reversible Protein Post‐Translational Modifications: Class D β‐Lactamase Carbamylation and Inhibition

**DOI:** 10.1002/chem.201902529

**Published:** 2019-08-20

**Authors:** Emma van Groesen, Christopher T. Lohans, Jürgen Brem, Kristina M. J. Aertker, Timothy D. W. Claridge, Christopher J. Schofield

**Affiliations:** ^1^ Department of Chemistry University of Oxford Oxford OX1 3TA UK; ^2^ Department of Biomedical and Molecular Sciences Queen's University Kingston ON K7L 3N6 Canada

**Keywords:** antibiotics, beta-lactamase, carbamylation, carbapenemase, NMR spectroscopy

## Abstract

Bacterial production of β‐lactamases with carbapenemase activity is a global health threat. The active sites of class D carbapenemases such as OXA‐48, which is of major clinical importance, uniquely contain a carbamylated lysine residue which is essential for catalysis. Although there is significant interest in characterizing this post‐translational modification, and it is a promising inhibition target, protein carbamylation is challenging to monitor in solution. We report the use of ^19^F NMR spectroscopy to monitor the carbamylation state of ^19^F‐labelled OXA‐48. This method was used to investigate the interactions of OXA‐48 with clinically used serine β‐lactamase inhibitors, including avibactam and vaborbactam. Crystallographic studies on ^19^F‐labelled OXA‐48 provide a structural rationale for the sensitivity of the ^19^F label to active site interactions. The overall results demonstrate the use of ^19^F NMR to monitor reversible covalent post‐translational modifications.

Bacterial resistance threatens the use of all antibacterials, including the clinically important β‐lactams.^1^ The class D serine β‐lactamases (SBLs) are of particular importance due to their widespread distribution and ability to degrade carbapenems, which are often antibiotics of last resort.^2^ Unlike other classes of SBLs, class D catalysis requires carbamylation of a lysine residue, which occurs through the reversible reaction of carbon dioxide/bicarbonate with the lysine *ϵ*‐amino group (Figure 1 A). As carbamylation is essential for class D SBL catalysis, it represents a potential target for inhibition. However, monitoring of reversible protein modifications (such as carbamylation) in solution is often challenging with currently available techniques. We report the application of ^19^F NMR spectroscopy to investigate carbamylation of the class D SBL OXA‐48, a major clinical cause of carbapenem resistance. We applied the method to monitor the binding interactions between OXA‐48 and the latest generation of SBL inhibitors, including avibactam and vaborbactam.^3^, ^4^ Our results show that ^19^F NMR may be generally applicable for the study of carbamylation in other proteins.

**Figure 1:**
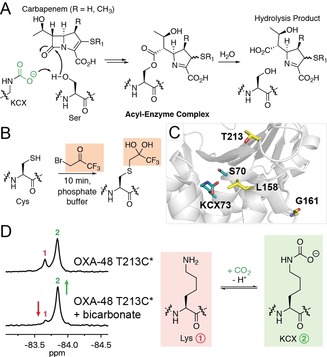
Carbapenemase mechanism and detection of carbamylation with ^19^F NMR. (A) Scheme showing carbapenem antibiotic degradation by a class D serine β‐lactamase, highlighting the role of the carbamylated lysine (KCX) as a general base. The carbamylated lysine is proposed to activate the “hydrolytic” water molecule. (B) Strategy for ^19^F‐labelling, wherein a cysteine residue is modified with 1‐bromo‐3,3,3‐trifluoroacetone. The hydrated form of the label was observed crystallographically (see below). (C) View from the active site of an OXA‐48 crystal structure (PDB 3HBR),^8^ showing the positions of Leu158, Gly161, and Thr213 (which were substituted with Cys residues and ^19^F‐labelled) relative to the carbamylated lysine KCX73. (D) ^19^F NMR spectra showing the impact of added sodium bicarbonate on the carbamylation state of ^19^F‐labelled OXA‐48 T213C*. The resonances at −83.66 ppm (1) and −83.84 ppm (2) were assigned as corresponding to the uncarbamylated (red) and carbamylated (green) states, respectively.

Due to the drawbacks encountered while using protein‐observe ^13^C NMR to monitor the carbamylation state of class D SBLs, such as poor sensitivity and the need for long acquisition times,^5^ we considered the use of ^19^F NMR, which we have applied to study loop movements in metallo‐β‐lactamases.^6^, ^7^ In previous studies, 1‐bromo‐3,3,3‐trifluoroacetone (BTFA) was used to selectively label cysteine residues introduced on these loops through mutagenesis (Figure 1 B).^6^, ^7^ We anticipated that a ^19^F label situated near the active site of OXA‐48, which has no cysteine residues in its wild‐type sequence, would be sensitive to its carbamylation state, so enabling the factors influencing this post‐translational modification to be investigated by ^19^F NMR spectroscopy. We proposed that the high sensitivity of ^19^F NMR, enhanced by the degeneracy of the three ^19^F nuclei in the BTFA‐derived label, could overcome the limitations associated with other methods for monitoring carbamylation in solution.

Identification of an appropriate position for ^19^F‐labelling was based on the consideration that the label must be sensitive to lysine carbamylation, whilst minimally impacting carbamylation and catalysis. Several OXA‐48 residues were identified as candidates for replacement with cysteine, based on their proximity to the carbamylated lysine as observed in crystallographic studies.^8^ Thus, the OXA‐48 I74C, V120C, L158C, G161C, and T213C variants were produced and purified (Figure 1 C and S1 in the Supporting Information). Complete labelling of the L158C, G161C, and T213C variants with BTFA was observed by mass spectrometry (within detection limits) under the conditions used (Figure S2). The ^19^F‐labelled enzymes are hereafter referred to as OXA‐48 L158C*, G161C*, and T213C*. Labelling of OXA‐48 I74C and V120C was not observed under these conditions, suggesting that the cysteine residues in these variants are inaccessible to the labelling reagent.

Following initial titration experiments to identify suitable enzyme concentrations (Figure S3), ^19^F NMR spectra for OXA‐48 L158C* and T213C* manifested two protein‐derived signals, while only a single signal was observed for OXA‐48 G161C* (Figure 1 D, S4). Addition of sodium bicarbonate to OXA‐48 L158C* and T213C* resulted in a change in the relative intensities of these two signals (Figure 1 D, S4), suggesting that they correspond to the carbamylated and uncarbamylated states of the enzyme. These spectra also suggest that OXA‐48 T213C* is carbamylated to a greater extent than OXA‐48 L158C* without addition of sodium bicarbonate. Based on kinetic studies (Table S1), the *k*_cat_ values determined for OXA‐48 T213C* with meropenem and nitrocefin were similar (but not identical) to wild‐type OXA‐48, while the activity of OXA‐48 L158C* was relatively poor. However, the *K*_m_ values for meropenem and nitrocefin were greater for OXA‐48 T213C* compared to wild‐type OXA‐48. The product profile of OXA‐48 T213C* with meropenem resembled what was observed for wild‐type OXA‐48, with both enzymes producing a similar ratio of carbapenem‐derived hydrolysis and lactone products (Figure S5).^9^

Circular dichroism analyses indicate that the secondary structure of both variants resembles that of wild‐type enzyme (Figure S6). Substitution of Leu158 appears to have a deleterious effect on enzyme activity and carbamylation, as manifested clearly in ^19^F NMR spectra acquired at lower pH (Figure S7). Although only a single ^19^F signal was observed for OXA‐48 G161C*, ^13^C NMR spectra with ^13^C‐labelled sodium bicarbonate confirm that carbamylation occurs for all three ^19^F‐labelled variants (Figure S8), suggesting that the chemical shift of the ^19^F label of OXA‐48 G161C* may be insensitive to the enzyme carbamylation state, and hence is not useful for monitoring carbamylation. Thr213, which is positioned on the β5‐β6 loop, is poorly conserved among class D SBLs (Figure S9). As OXA‐48 T213C* was judged to more closely resemble the wild‐type enzyme (although, as noted, the ^19^F‐labelling did apparently impact kinetic behaviour), it was chosen for subsequent ^19^F NMR studies.

To validate the use of ^19^F NMR to study carbamylation, we compared results obtained with OXA‐48 T213C* to previous observations made using ^13^C NMR (and NaH^13^CO_3_) to examine the carbamylation state of reversible covalent and non‐covalent OXA enzyme complexes (with avibactam and halide ions).^5^ We have previously observed that avibactam, a diazabicyclooctane (DBO)‐type SBL inhibitor which forms a reversible covalent complex (Figure 2 A),^10^ disfavours, but does not ablate carbamylation of OXA enzymes.^5^ The extent of carbamylation of the avibactam:enzyme complex is further decreased in the presence of halide ions, which also inhibit OXA enzymes, likely by decarbamylating the acyl‐enzyme complex formed during substrate hydrolysis.^5^

**Figure 2:**
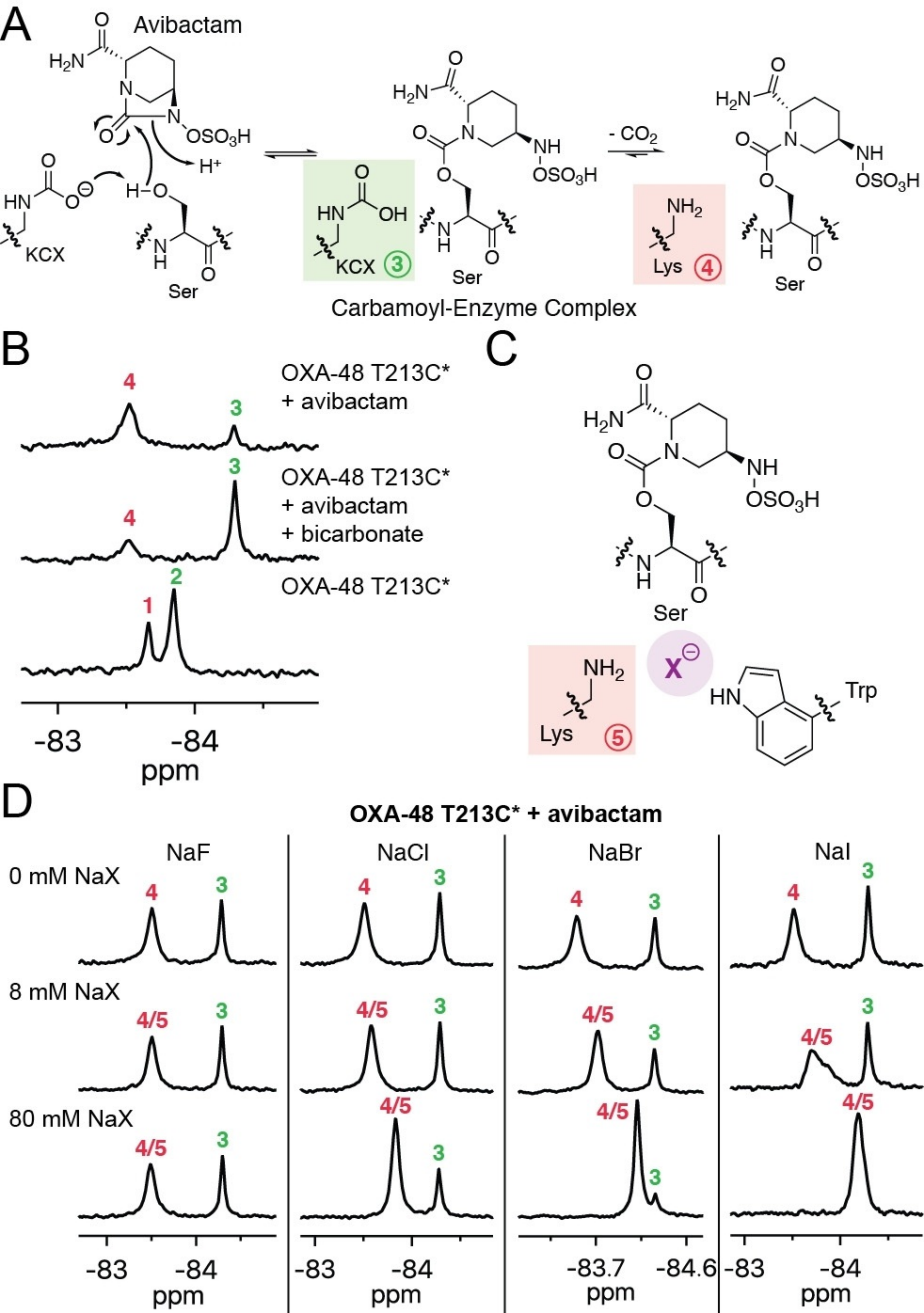
^19^F NMR studies on the interaction of OXA‐48 T213C* with avibactam and halide ions. (A) Proposed interaction of avibactam with a class D serine β‐lactamase, showing the carbamylated (green) and uncarbamylated (red) states of the complex. (B) ^19^F NMR spectra showing the impact of avibactam on the carbamylation state of OXA‐48 T213C* with and without added sodium bicarbonate. (C) Proposed binding mode of a halide ion in the uncarbamylated active site of the OXA‐48:avibactam complex, based on previous crystallographic work.^5^ (D) ^19^F NMR spectra showing the impact of halide ions on the carbamylation state and ^19^F chemical shifts of OXA‐48 T213C*. Note that the addition of sodium fluoride may also affect the pH of the sample. Trifluoroethanol was used as an internal standard for ^19^F NMR, and CFCl_3_ in CDCl_3_ was used as an external standard.

Following treatment of OXA‐48 T213C* with avibactam, new ^19^F NMR signals were observed at −83.50 ppm and −84.28 ppm (Figure 2 B). Addition of bicarbonate impacted on the relative levels of these two signals, suggesting that they correspond to the uncarbamylated and carbamylated states of the covalent avibactam‐enzyme complex, respectively. Furthermore, these spectra show that carbamylation of the avibactam‐derived OXA‐48 T213C* complex is disfavoured compared to OXA‐48 T213C* alone. The nucleophilic serine of OXA‐48 appears to hydrogen bond with the carbamylated lysine, which may contribute to its stability;^8^ covalent modification of the serine with avibactam (or a substrate) apparently removes this hydrogen bond interaction, such that carbamylation is relatively disfavoured in the avibactam‐enzyme complex.^5^ The impact of avibactam as observed by ^19^F NMR was apparently greater than what was observed by ^13^C NMR.^5^ While the ^19^F NMR experiments do not require any bicarbonate, NaH^13^CO_3_ is required for the ^13^C NMR experiments, and this added bicarbonate may mask subtle changes in carbamylation levels. The SBL inhibitors zidebactam and relebactam, which are DBOs structurally related to avibactam, also favoured decarbamylation of their respective covalent complexes formed with OXA‐48 T213C* (Figure S10).

As described above, class D SBLs are inhibited by halide ions, which are proposed to promote decarbamylation of the acyl‐enzyme complex.^5^ The impact of halide ions on carbamylation was investigated with OXA‐48 T213C*, using the stable avibactam‐derived complex as a model for the acyl‐enzyme complex. While chloride, bromide, and iodide ions had little impact on the ^19^F NMR signals of OXA‐48 T213C* in the absence of avibactam (Figure S11), these halide ions decreased the extent of carbamylation of the OXA‐48 complex derived from avibactam (Figure 2 C, 2D). Furthermore, the extent of decarbamylation appears to correlate with the size of the halide ion, consistent with ^13^C NMR results.^5^ Notably, the ^19^F signal corresponding to the decarbamylated avibactam‐enzyme complex shifted according to the halide ion concentration (Figure 2 D). This indicates that the reversible binding of halide ions to this complex occurs under fast exchange conditions, relative to the NMR timescale. By contrast, no impact was observed on the ^19^F signal corresponding to the carbamylated enzyme, indicating halide ions preferentially bind to the uncarbamylated active site.

Following validation of the method, we next used the ^19^F NMR method to investigate the impact of temperature on the extent of carbamylation. As the temperature of OXA‐48 T213C* was increased, the extent of carbamylation was observed to decrease (Figure 3 A). However, carbamylation was restored upon lowering the temperature (data not shown), suggesting that the carbon dioxide levels in the solution were not (irreversibly) depleted at higher temperatures. The impact of temperature on the OXA‐48 T213C* carbamylation state is greater than the expected impact of temperature on carbon dioxide solubility;^11^ pH effects (e.g., the Bohr effect) related to carbon dioxide levels and temperature^12^ are expected to be small due to sample buffering. At higher temperatures, the extent of carbamylation of the avibactam‐derived OXA‐48 T213C* complex was observed to decrease more substantially relative to the unmodified enzyme (Figure 3 A). Similar temperature‐dependent effects were observed for the complexes derived from the DBO inhibitors zidebactam and relebactam (Figure S12). Thus, temperature should be a consideration when examining the carbamylation status of class D SBLs, and by implication, other proteins.

**Figure 3:**
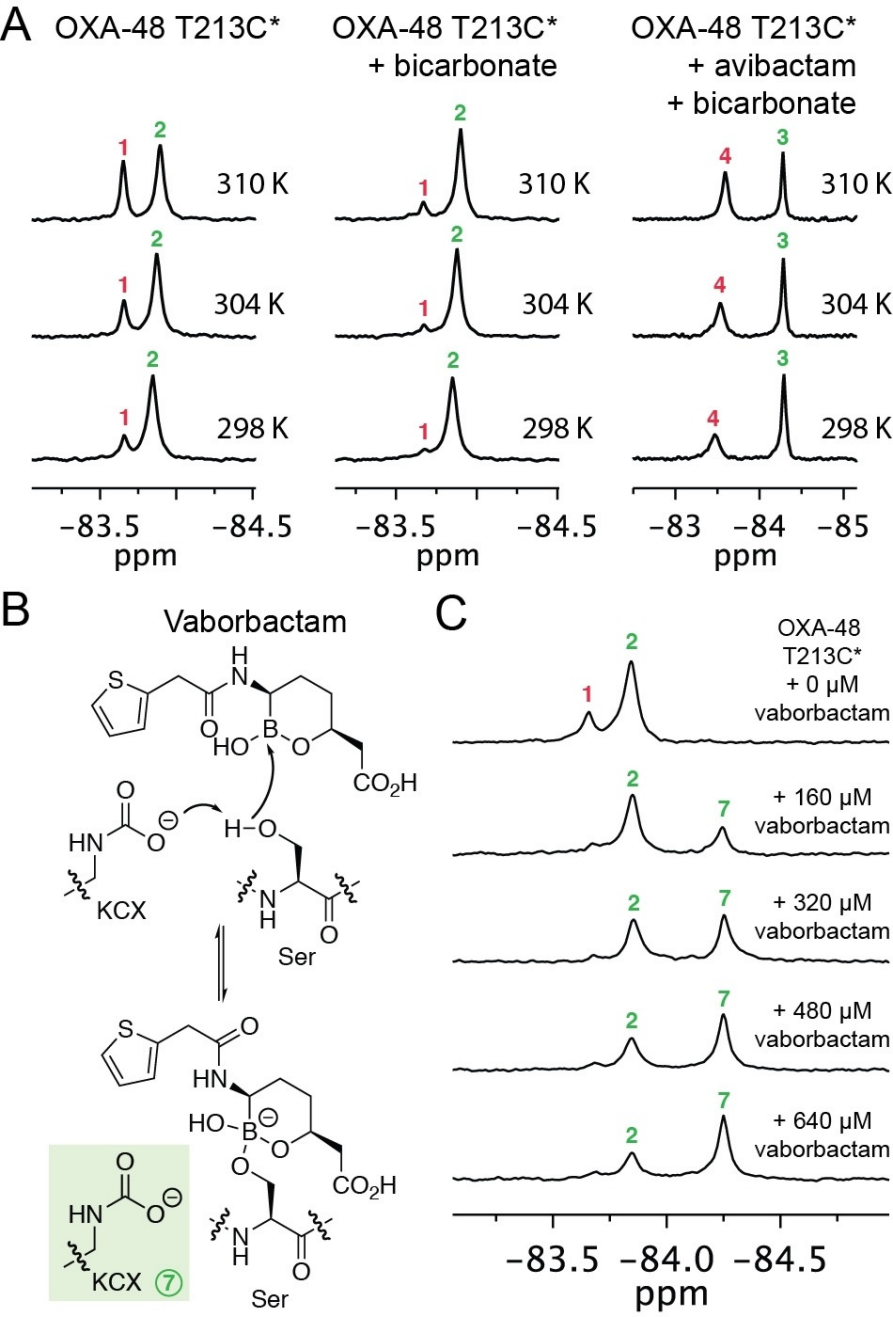
^19^F NMR studies on the impact of temperature and boronate binding on OXA‐48 T213C*. (A) ^19^F NMR spectra showing the impact of temperature on the carbamylation state of OXA‐48 T213C* alone, with bicarbonate, and with avibactam and bicarbonate. (B) Proposed interaction of vaborbactam with a class D serine β‐lactamase to form a tetrahedral complex. Based on ^13^C NMR studies, the lysine residue in this complex is expected to be carbamylated (Figure S14).^13^, ^15^ (C) ^19^F NMR spectra showing the titration of OXA‐48 T213C* (160 μm) with vaborbactam, resulting in a new ^19^F signal (7). From these data, the binding affinity for vaborbactam with OXA‐48 T213C* could be determined (Figure S15).

Cyclic boronates are currently of considerable interest as inhibitors of SBLs, metallo‐β‐lactamases (MBLs), and possibly of penicillin‐binding proteins (PBPs; the bacterial target of the β‐lactam antibiotics) (Figure 3 B).^13^−^15^ Vaborbactam (formerly RPX‐7009), a (predominantly) monocyclic boronate β‐lactamase inhibitor, was recently FDA approved for use in combination with the carbapenem meropenem.^4^ Boronates form covalent complexes with SBLs in which the nucleophilic serine is bonded to the tetrahedral boron, mimicking the tetrahedral complex formed during β‐lactamase catalysis.^13^, ^15^

Following addition of vaborbactam to OXA‐48 T213C*, a new ^19^F signal appeared, assigned as corresponding to formation of the boronate‐enzyme complex (Figure 3 B, 3 C). While these spectra alone cannot differentiate between covalent and non‐covalent binding, crystallographic studies of vaborbactam with SBLs AmpC and CTX‐M‐15 indicate that a covalent interaction with OXA‐48 is likely.^14^ Addition of bicarbonate to the complex did not result in any observed change in the ^19^F spectrum (Figure S13); consistent with this, ^13^C NMR spectra using NaH^13^CO_3_ indicate that carbamylation is maintained in the vaborbactam‐enzyme complex (Figure S14), similar to what has been observed for cyclic boronates and class D SBLs.^13^, ^15^ While reaction with avibactam may disfavour carbamylation by removing a hydrogen bond interaction involving the nucleophilic serine, it is not clear why the same is not true for vaborbactam; however, crystallographic studies of the class D SBL OXA‐10 with cyclic boronate 1C^13^ suggest that hydrogen bond interactions occurring in the sp^3^‐hybridized boronate complex could stabilize lysine carbamylation despite the covalent modification of serine. Titration experiments show that vaborbactam does not interact strongly with OXA‐48, consistent with reported inhibition studies (Figure 3 C, S15).^16^ In agreement, ^19^F NMR enabled the strength of the binding interaction to be investigated quantitatively, yielding a *K*_D_ of ≈470 μm for vaborbactam with OXA‐48 T213C* (Figure S15).

The sensitivity of the ^19^F label on Cys213 to the carbamylation state of OXA‐48 T213C* was of interest, given the apparent observed distance between Thr213 and the carbamylated lysine (11.5 Å between the Thr213 C‐3 oxygen and N^*ϵ*^ of KCX73 in PDB entry 3HBR; Figure 1 C).^8^ To provide a structural rationale for this sensitivity, crystallographic studies were carried out. A structure of OXA‐48 T213C* was solved by molecular replacement, using PDB 4S2P as a search model (Figure 4 A, Table S2).^17^ The overall fold of OXA‐48 T213C* (PDB entry 6RJ7) was the same as that observed for the wild‐type enzyme (e.g., root mean square deviation of 0.613 Å for backbone Cα atoms as compared to PDB 3HBR),^8^ and only minor deviations were observed in the region bearing the ^19^F label. Notably, unlike our crystal structure of 1,1,1‐trifluoroacetone (TFA)‐labelled metallo‐β‐lactamase SPM‐1,^18^ clear electron density representing the TFA label was observed in the OXA‐48 T213C* structure (Figure 4). The trifluoroacetone label is present in its hydrated (diol) form in the crystalline state (Figure 4 B). This is consistent with small molecule hydration studies on trifluoromethyl ketones.^19^ Hence, our ^19^F NMR studies in solution likely represent, at least normally, the hydrated state of the TFA label.

**Figure 4:**
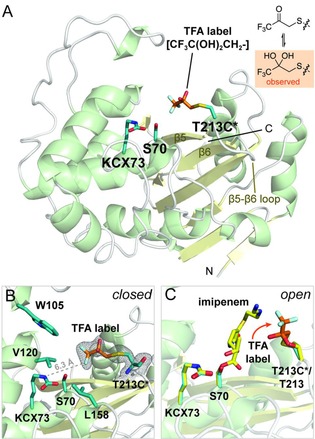
Crystallographic studies of OXA‐48 T213C*. (A) Views from a crystal structure of OXA‐48 T213C* (PDB 6RJ7). The 1,1,1‐trifluoroacetone (TFA) label is shown with orange sticks, while Cys213, Ser70, and the carbamylated lysine KCX73 are shown with blue sticks. (B) Orientation of the TFA label towards the hydrophobic pocket made up of Trp105, Val120, and Leu158 (blue sticks), likely explaining the sensitivity of the label to the carbamylation status of Lys73. The electron density corresponding to the hydrated TFA label is represented as a 2mFo‐DFc map, contoured at 1σ. (C) Overlay of the structure of the imipenem‐derived OXA‐48 complex (yellow sticks; PDB 5QB4)^20^ with the structure of OXA‐48 T213C* (cartoon, blue and orange sticks). The crystallographically observed position of the TFA label in the overlay was adjusted to represent the expected movement of the label resulting from substrate or inhibitor binding, as observed through changes in the ^19^F NMR spectrum (Figure 2 B, 3 C).

Comparison of the OXA‐48 T213C* structure with the structure of a carbapenem‐derived OXA‐48 acyl‐enzyme complex^20^ implies the overlap of the TFA label and elements of the carbapenem acyl‐enzyme complex (Figure 4 C). Consequently, the ^19^F label must be displaced from this position following substrate or inhibitor binding, as manifested by changes in the ^19^F NMR spectrum (Figure 2 B, 3 C). As above, it is unclear whether the observed chemical shift differences relate to conformational changes associated with inhibitor binding, or if other interactions also contribute to the observed differences. The presence of the ^19^F label in OXA‐48 T213C* close to the active site likely causes the observed changes in kinetic properties when compared to wild‐type OXA‐48 (Table S1). However, since these differences are relatively minor, and as the carbamylation of the ^19^F‐labelled enzyme behaves similarly to what was previously observed for wild‐type OXA‐48,^5^ the presence of the label does not appear to have a major impact on carbamylation and enzymatic activity, suggesting that the positioning of the label in OXA‐48 T213C* is dynamic, and does not obstruct access to the active site.

^19^F NMR is a powerful approach for monitoring reversible post‐translational modifications, as demonstrated through our studies on the factors influencing the carbamylation state of the clinically important SBL OXA‐48.^22^ The high sensitivity of ^19^F NMR overcomes some drawbacks associated with other methods for investigating carbamylation, including ^13^C NMR.^5^ The role of carbamylation in modulating enzyme inhibition is largely unexplored, with one notable exception being the class D SBLs, which are of major clinical relevance. The ^19^F‐based methodology reported here will enable the role of carbamylation in interactions between these clinically important carbapenemases and SBL inhibitors to be further investigated, providing mechanistic information to guide the design of new generations of inhibitors.

Recent work has indicated that the number of proteins that contain carbamylated lysine residues has been substantially underestimated.^23^ Indeed, it is likely that the full scope of reversible post‐translational modifications induced by small molecules is far from being fully appreciated. There is growing research interest in reversible post‐translational carbamylation in the regulation of protein function (as classically shown for haemoglobin) and stability.^24^ ^19^F NMR (using labels introduced by post‐translational modification, or by de novo incorporation of ^19^F‐labelled amino acids)^25^, ^26^, ^27^ will enable further detailed mechanistic investigations into protein carbamylation and its regulation by factors including temperature and pH. It should be noted, however, that our studies highlight the importance of establishing that the properties of the wild‐type enzyme are adequately represented by the ^19^F‐labelled variant. ^19^F NMR also demonstrates clear promise for investigating other reversible covalent and non‐covalent (e.g., metal ion and gas binding) post‐translational modifications.^6^

## Conflict of interest

The authors declare no conflict of interest.

## Supporting information

As a service to our authors and readers, this journal provides supporting information supplied by the authors. Such materials are peer reviewed and may be re‐organized for online delivery, but are not copy‐edited or typeset. Technical support issues arising from supporting information (other than missing files) should be addressed to the authors.

Supplementary
