# Supplementary material for: 19F NMR Monitoring of Reversible Protein Post‐Translational Modifications: Class D β‐Lactamase Carbamylation and Inhibition
Source: Chemistry. 2019 Aug 20;25(51):11837–41. doi: 10.1002/chem.201902529 (PMC6771976; doi:10.1002/chem.201902529)
Supplement: Supplementary file 1 — Supplementary [file CHEM-25-11837-s001.pdf]

CHEMISTRY
A European Journal

Supporting Information

^{19}F NMR Monitoring of Reversible Protein Post-Translational Modifications: Class D β-Lactamase Carbamylation and Inhibition

Emma van Groesen+,[a] Christopher T. Lohans+,*[a, b] Jürgen Brem+,[a] Kristina M. J. Aertker,[a] Timothy D. W. Claridge,[a] and Christopher J. Schofield*[a]

chem_201902529_sm_miscellaneous_information.pdf

Author Contributions

K.A. Formal analysis: Supporting; Investigation: Supporting.

Supporting Information Contents

Experimental	S3
Fig. S1. Crystal structure showing the positions tested for ^{19}F-labelling.	S7
Fig. S2. Mass spectrometric analysis of BTFA labelling of OXA-48 variants	S8
Fig. S3. ^{19}F-NMR spectra for varying concentrations of OXA-48 T213C*	S9
Fig. S4. ^{19}F-NMR spectra showing impact of bicarbonate on OXA-48 variants	S10
Table S1. Michaelis-Menten kinetic values for ^{19}F-labelled OXA-48 variants	S11
Fig. S5. Product profiles of OXA-48 and OXA-48 T213C* with meropenem	S12
Fig. S6. Circular dichroism spectra for ^{19}F-labelled OXA-48 variants	S13
Fig. S7. Impact of pH on the carbamylation state of ^{19}F-labelled OXA-48 variants	S14
Fig. S8. ^{13}C-NMR spectra of ^{19}F-labelled OXA-48 variants	S15
Fig. S9. Amino acid sequence alignment of class D SBLs	S16
Fig. S10. Interaction of OXA-48 T213C* with diazabicyclooctane inhibitors	S17
Fig. S11. Impact of halide ions on the ^{19}F-NMR spectrum of OXA-48 T213C*	S18
Fig. S12. Impact of temperature on carbamylation of OXA-48 T213C* with DBOs	S19
Fig. S13. Impact of bicarbonate on the vaborbactam OXA-48 T213C* complex	S20
Fig. S14. ^{13}C-NMR spectra of OXA-48 T213C* with vaborbactam	S21
Fig. S15. Binding curve for vaborbactam with OXA-48 T213C*	S22
Table S2. Processing and refinement statistics for OXA-48 T213C* crystallography	S23
References	S24

Experimental

General

Unless otherwise stated, all chemicals were from Sigma-Aldrich. Avibactam was from AstraZeneca, relebactam and zidebactam were from MedKoo Biosciences, meropenem was from Glentham Life Sciences, and vaborbactam was from Cambridge Bioscience.

Protein Production and BTFA Labelling

The plasmids encoding for OXA-48 I74C, V120C, L158C, G161C, and T213C were prepared by site-directed mutagenesis according to the QuikChange protocol (Agilent), using pNIC28-Bsa4-OXA-48 as a template.[1] *Escherichia coli* BL21(DE3) cells were transformed with the plasmids encoding these OXA-48 variants. Transformants were grown overnight in 100 mL 2TY media with 50 μg/mL kanamycin at 37 °C and 180 rpm. The overnight cultures were used to inoculate (1 %) 2TY media (3 L, supplemented with 50 μg/mL kanamycin), which was incubated at 37 °C and 180 rpm. Once the optical density (600 nm) of the culture reached 0.6, isopropyl β-D-thiogalactopyranoside (IPTG) was added to a final concentration of 0.5 mM, and the culture was incubated overnight at 18 °C and 180 rpm. Cells were harvested by centrifugation (11,000 x g, 10 min), frozen on dry ice, and stored at -80 °C.

The frozen cells were re-suspended in HisTrap Buffer A (50 mM HEPES, pH 7.5, 500 mM NaCl, 20 mM imidazole) supplemented with DNase I, lysed by sonication, and centrifuged (32,000 x g, 30 min). The supernatant was passed through 0.45 μm filters, loaded onto a 5 mL HisTrap HP column (GE Life Sciences) pre-equilibrated with HisTrap Buffer A, and washed with 20 column volumes of HisTrap Buffer A. Protein was eluted with a linear gradient of HisTrap Buffer A and HisTrap Buffer B (50 mM HEPES, pH 7.5, 500 mM NaCl, 500 mM imidazole) over 20 min (from 0 % to 100 % Buffer B), and 5 mL fractions were collected. Fractions containing eluted protein

were combined, concentrated (using 10 kDa molecular-weight cut-off Amicon centrifugal filters; EMD Millipore), and loaded onto a 300 mL Superdex 200 column (GE Life Sciences) pre-equilibrated in gel filtration buffer (50 mM HEPES, pH 7.5, 200 mM NaCl). The Superdex column was washed with one column volume of gel filtration buffer, and 5 mL fractions were collected. Fractions of the desired purity (>95%), as identified by SDS-PAGE, were combined, buffer exchanged into 50 mM sodium phosphate, pH 7.5, concentrated using an Amicon centrifugal filter, frozen in liquid nitrogen, then stored at -80 °C. The identity and purity of the purified protein was confirmed by MS.

For 1-bromo-3,3,3-trifluoroacetone (BTFA) labelling,[2] protein aliquots were thawed on ice; neat BTFA was added to give a final ratio of 15 BTFA : 1 enzyme. The mixture was incubated on ice for 10 minutes, then buffer exchanged into 50 mM sodium phosphate, pH 7.5 using a pre-equilibrated PD-10 column (GE Healthcare), then dialyzed overnight against 50 mM sodium phosphate, pH 7.5 using a Slide-A-Lyzer Dialysis cassette (10 kDa molecular-weight cut-off filter). The labelled protein was concentrated using an Amicon centrifugal filter, frozen in liquid nitrogen, and stored at -80 °C. Complete labelling (within detection limits) was confirmed by mass spectrometry (Figure S2).

NMR Spectroscopy

^{1}H, ^{13}C, and ^{19}F NMR spectra were acquired using a Bruker AVIII HD 600 MHz spectrometer equipped with a 5 mm BB-F/^{1}H Prodigy N_2 cryoprobe. Unless stated otherwise, samples were prepared in 50 mM sodium phosphate, pH 7.5, 10 % D_2O; spectra were acquired at 298 K. 1H NMR spectra consisted of 32 scans, and were acquired with an acquisition time of 1.70 s, a relaxation delay of 1 s, and a spectral width of 16.02 ppm. ^{13}C-NMR spectra consisted of 2048 scans, and were acquired with an acquisition time of 0.91 s, a relaxation delay of 2 s, and a spectral

width of 238.90 ppm. ^{19}F-NMR spectra consisted of 512 scans, and were acquired with an acquisition time of 0.49 s, a relaxation delay of 2 s, and with a spectral width of 237.18 ppm. Unless stated otherwise, 160 μM ^{19}F-labelled protein was used in all experiments. Trifluoroethanol (TFE) was used as an internal standard, referenced at -76.62 ppm; 0 ppm was defined using $CFCl_3$ in $CDCl_3$ as an external standard. A line broadening of 10 Hz was applied to ^{13}C- and ^{19}F-NMR spectra.

Mass Spectrometry

Protein mass spectra were acquired using a Waters Micromass LCT Premier XE spectrometer coupled with an Acquity UPLC system. Samples consisted of 1 μM protein prepared in 50 mM sodium phosphate, pH 7.5. Protein signals were deconvoluted using the MaxEnt1 function of MassLynx V4.1 (Waters).

Circular Dichroism

Circular dichroism spectra were acquired at 25 °C using a Chirascan CD spectrometer (Applied Photophysics model).[3] Samples were made up of 0.2 mg/mL enzyme in 10 mM sodium phosphate, pH 7.5, and a 0.1 cm cuvette was used. The data shown represent the smoothed average of three measurements over the range of 185 nm to 260 nm, using 0.5 nm increments. Each point was averaged for 1 s.

Enzyme Kinetics

Enzymatic hydrolysis of nitrocefin and meropenem was monitored using a PHERAstar FS plate reader (BMG Labtech).[3] Hydrolysis experiments were performed at 25 °C with 96-well UV-Star Microplates (Greiner Bio-One), monitoring the change in absorbance at 486 nm for nitrocefin (ε = 20,500 M^{-1} cm^{-1}), or at 295 nm for meropenem (ε = 10,940 M^{-1} cm^{-1}). The buffer used for assays consisted of 100 mM sodium phosphate, pH 7.5, 50 mM sodium bicarbonate with 0.01%

Triton X-100. For the meropenem degradation assays, enzyme concentrations of 75 nM (OXA-48 wild-type), 2500 nM (OXA-48 L158C*) or 400 nM (OXA-48 T213C*) were used. For the nitrocefin degradation assays, enzyme concentrations of 25 pM (OXA-48 wild-type), 125 pM (OXA-48 L158C*), or 50 pM (OXA-48 T213C*) were used. Non-linear regression analyses were carried out using Prism 5 (GraphPad) to determine kinetic parameters.

X-ray Crystallography

Purified and ^{19}F-labelled OXA-48 T213C* was buffer exchanged into 50 mM MES, pH 6.0, with 50 mM $NaHCO_3$. Crystallisation trials were carried out using the PACT premier broad screen (Molecular Dimensions). A structure of OXA-48 T213C* (PDB 6RJ7) was solved using a crystal obtained from a well solution consisting of 0.2 M $CaCl_2$, 0.1 M Tris, pH 8.0, and 20 % w/v PEG 6000. Data were collected at the Diamond Light Source synchrotron IO4-1 beamline at 100 K, were indexed and integrated with XDS, and scaled using SHELX.[4] The structure was solved by molecular replacement using Phaser,[5] using PDB entry 4S2P as a search model.[6] Fitting and refinement were carried out using COOT[7] and PHENIX,[8] until R_{work} and R_{free} no longer converged. The statistics for data collection and refinement are described in Table S2.

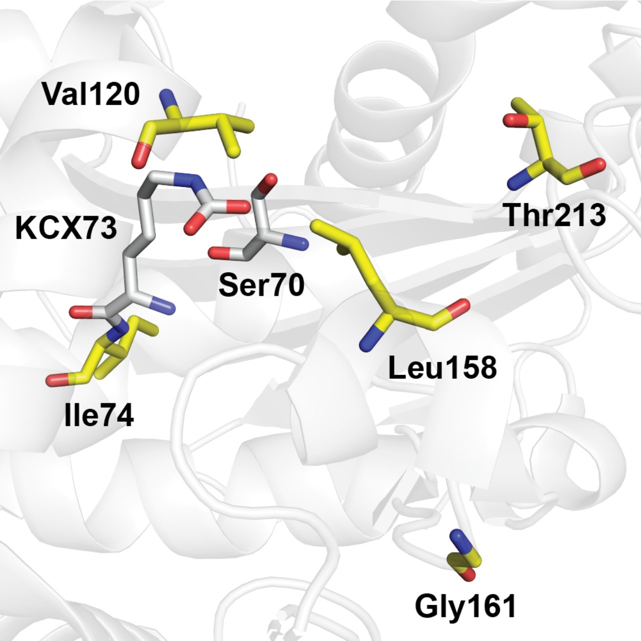

Figure S1. View from a crystal structure of OXA-48 (PDB 3HBR),[9] showing the positions chosen for ^{19}F-labelling with 1-bromo-3,3,3-trifluoroacetone. The nucleophilic serine, Ser70, and the carbamylated lysine, KCX73, are represented as white sticks; the residues that were substituted with cysteine residues are shown as yellow sticks.

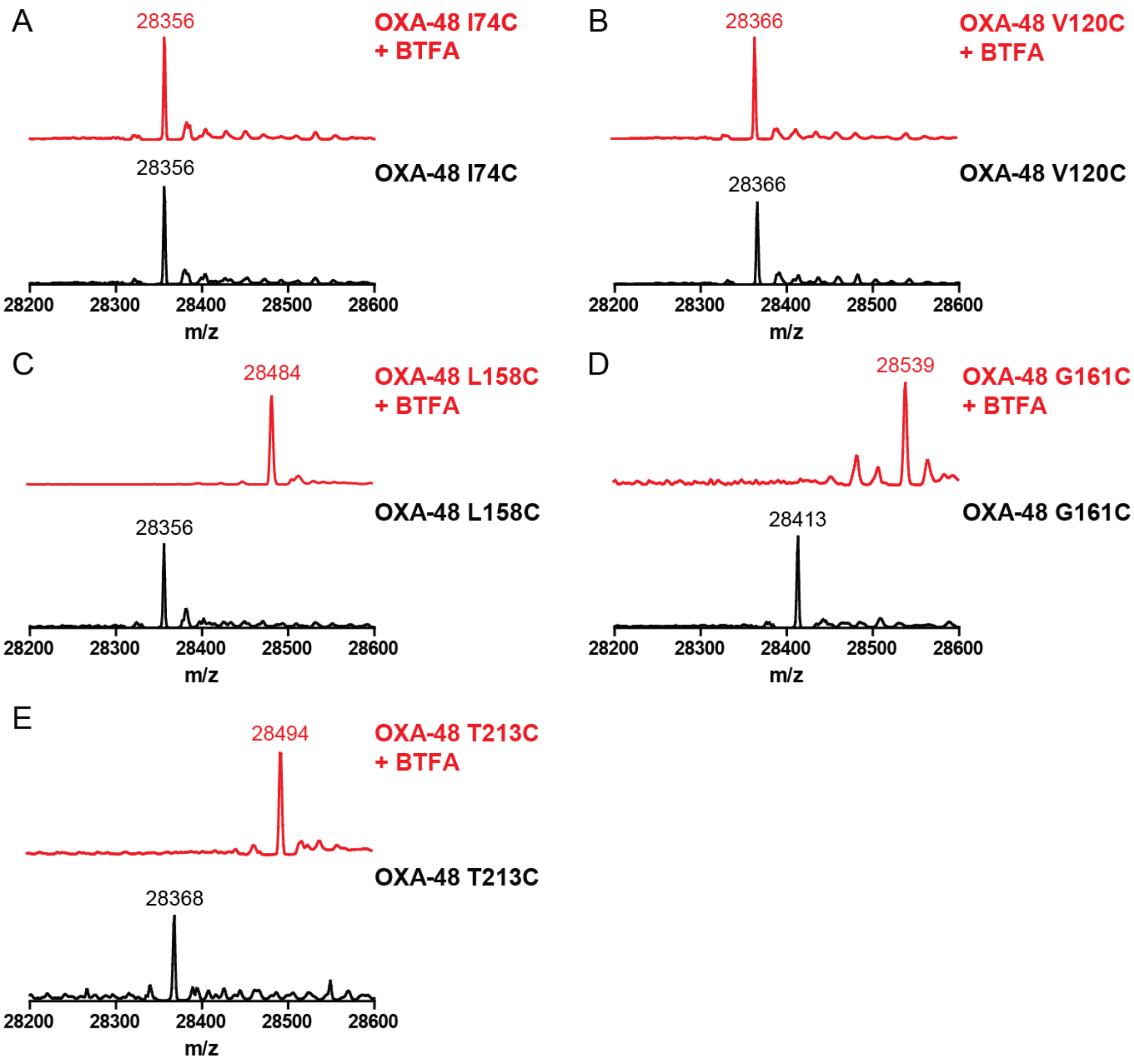

Figure S2. Mass spectrometric analysis of the BTFA labelling of OXA-48 variants. Mass spectra for the (A) OXA-48 I74C, (B) V120C, (C) L158C, (D) G161C, and (E) T213C variants, alone, or following treatment with 1-bromo-3,3,3-trifluoroacetone (BTFA). Complete apparent labelling (within detection limits) of OXA-48 L158C, G161C, and T213C by reaction with a single molecule of BTFA was observed under the conditions used (see Experimental Details). Masses: OXA-48 I74C (calculated 28,355 Da, observed m/z 28,356 Da), OXA-48 I74C + BTFA (calculated 28,465 Da, not observed), OXA-48 V120C (calculated 28,369 Da, observed m/z 28,366 Da), OXA-48 V120C + BTFA (calculated 28,479 Da, not observed), OXA-48 L158C (calculated 28,355 Da, observed m/z 28,356 Da), OXA-48 L158C + BTFA (calculated 28,465 Da, observed m/z 28,484 Da), OXA-48 G161C (calculated 28,411 Da, observed m/z 28,413 Da), OXA-48 G161C + BTFA (calculated 28,521 Da, observed m/z 28,539 Da), OXA-48 T213C (calculated 28,367 Da, observed m/z 28,368 Da), and OXA-48 T213C + BTFA (calculated 28,477 Da, observed m/z 28,494 Da). Note that the observed masses for BTFA-labelled OXA-48 L158C, T213C, and G161C were ~18 Da greater than calculated. This mass difference suggests that the ketone group in the label is (at least, predominantly) hydrated, as observed crystallographically (Figure 4), and consistent with small molecule hydration studies.[10]

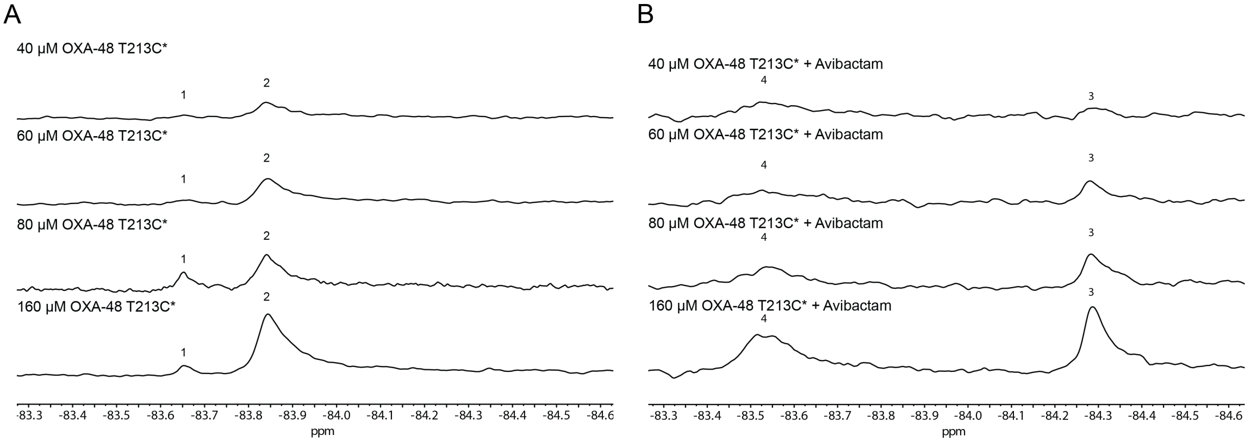

Figure S3. ^{19}F-NMR spectra for varying concentrations of OXA-48 T213C*. Spectra were acquired using 40, 60, 80, and 160 µM OXA-48 T213C* in 50 mM sodium phosphate, pH 7.5, 10% D_2O, (A) alone, and (B) with 160 µM avibactam. The numbers assigned to the peaks correspond to the assignments shown in Figures 1D and 2B. Under the conditions used (i.e., acquisition of 512 scans), the spectrum acquired using 160 µM enzyme gave a satisfactory signal intensity, whereas lower concentrations led to an insufficient signal/noise ratio. Hence, 160 µM enzyme was used for all subsequent ^{19}F-NMR experiments.

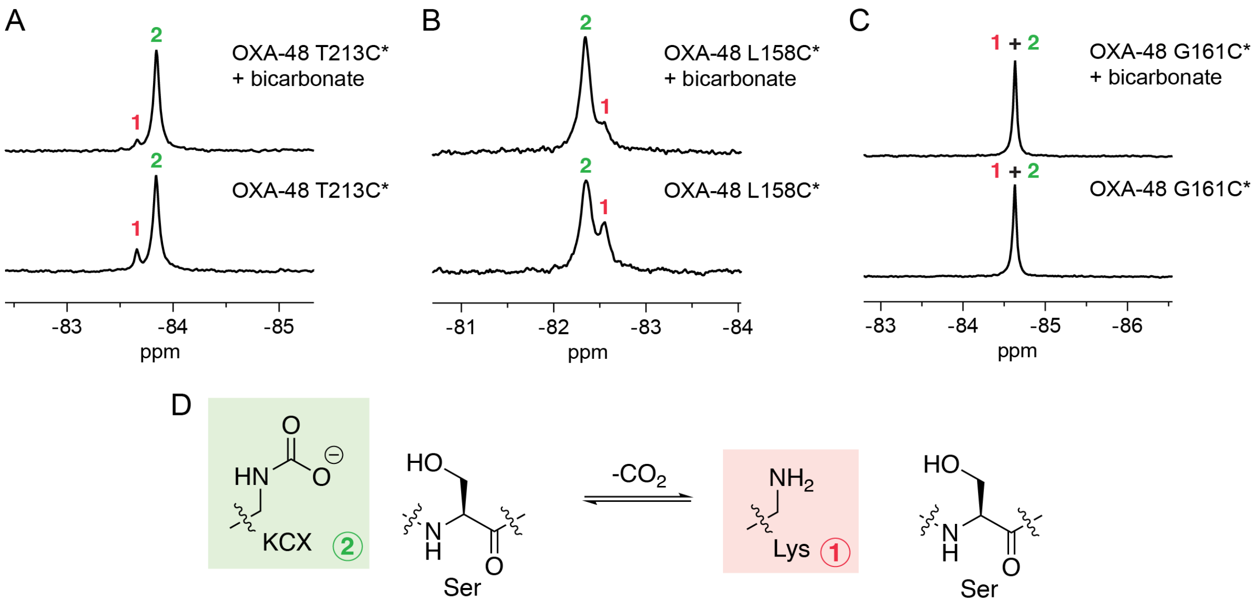

Figure S4. ^{19}**F-NMR spectra of** ^{19}**F-labelled OXA-48 variants with and without added bicarbonate.** ^{19}F-NMR spectra of 160 µM (A) OXA-48 T213C*, (B) OXA-48 L158C*, and (C) OXA-48 G161C*. Spectra were measured in 50 mM sodium phosphate, pH 7.5, 10 % D_2O, both with and without 1 mM sodium bicarbonate. These spectra show that the ^{19}F-label in OXA-48 T213C* and OXA-48 L158C* is sensitive to the enzyme carbamylation state, while the ^{19}F-label in OXA-48 G161C* is not. The positions of these residues relative to the carbamylated lysine KCX73 are shown in Figure S1. (D) Scheme showing the carbamylated and uncarbamylated states of a class D SBL. The numbers assigned to these states correspond to the ^{19}F signals labeled with these numbers in panels A, B, and C. Note that the relative vertical scales of the NMR spectra in panels A, B, and C are independent.

Table S1. Michaelis-Menten kinetic values for the ^{19}F-labelled OXA-48 variants.

Enzyme	Substrate	k_{cat} (s^{-1})	K_m (μM)	k_{cat}/K_m ($M^{-1}s^{-1}$) x 10^{-6}
**OXA-48 (wild-type)**	nitrocefin	663.2 ± 17.8	34.6 ± 3.7	19.1
	meropenem	0.057 ± 0.00094	<1.9[b]	>0.03
**OXA-48 T213C***	nitrocefin	838.6 ± 89.9	192.2 ± 42.7	4.36
	meropenem	0.089 ± 0.0022	11.7 ± 1.7	0.008
**OXA-48 L158C***	nitrocefin	101.68 ± 7.44	44.85 ± 11.77	2.3
	meropenem	0.013 ± 0.00044	24.4 ± 3.5	0.00053

[a]Assay conditions are described in the Experimental section.
[b]In this case, the K_m value could not be accurately measured due to experimental limitations, and so the value shown represents the smallest substrate concentration used for the kinetic determination.

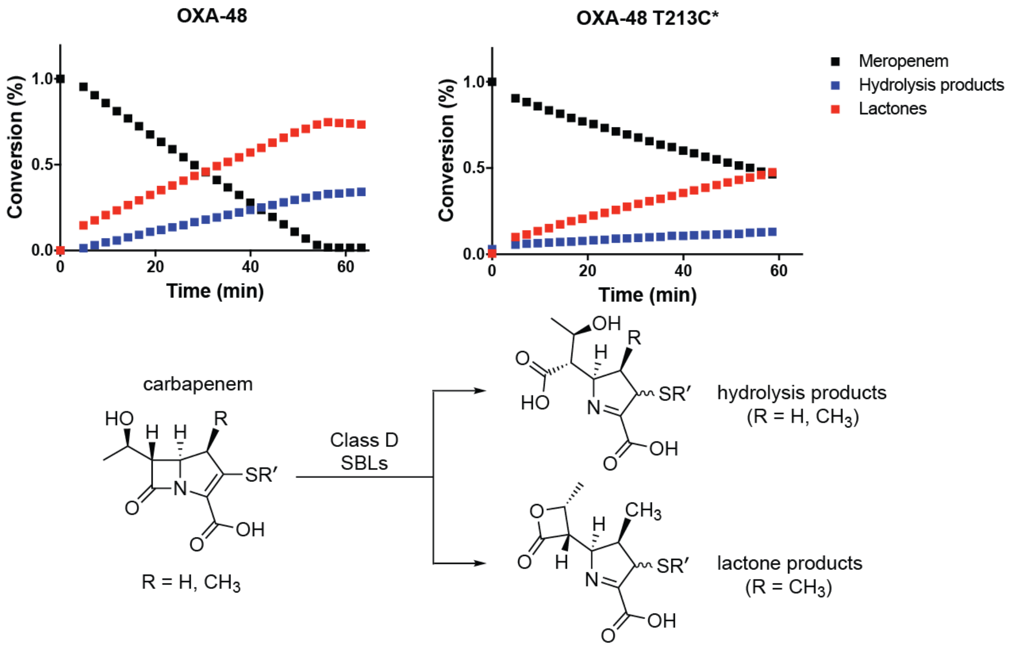

Figure S5. Product profiles of OXA-48 and OXA-48 T213C* with meropenem. NMR time courses for OXA-48 and OXA-48 T213C* (5 μM) with meropenem (1 mM) in 50 mM sodium phosphate, pH 7.5, 10 % D_2O, showing the levels of hydrolysis and lactone products formed over time. Although OXA-48 T213C* appears to be less active than the wild-type enzyme under the conditions used for NMR (contrasting the kinetic studies reported in Table S1, which differed in terms of the buffer conditions and enzyme concentrations used), these data show that the ratio of lactone and hydrolysis products are similar for both wild-type OXA-48 and OXA-48 T213C*. Note that the lactone and hydrolysis products are present as a mixture of tautomers and diastereomers.[11]

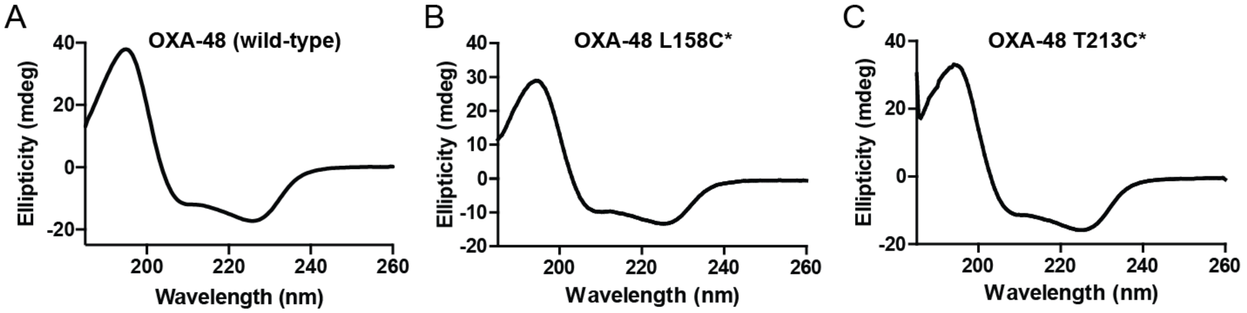

Figure S6. Circular dichroism spectra for the ^{19}F-labelled OXA-48 variants. The spectra acquired for the ^{19}F-labelled OXA-48 L158C* and T213C* variants resemble that of wild-type OXA-48. Based on these spectra (and as supported by the kinetic data shown in Table S1 and the crystallographic studies shown in Figure 4), the amino acid substitutions and ^{19}F-labelling do not appear to significantly impact on the overall folds of the OXA-48 variants, as compared to the wild-type enzyme.

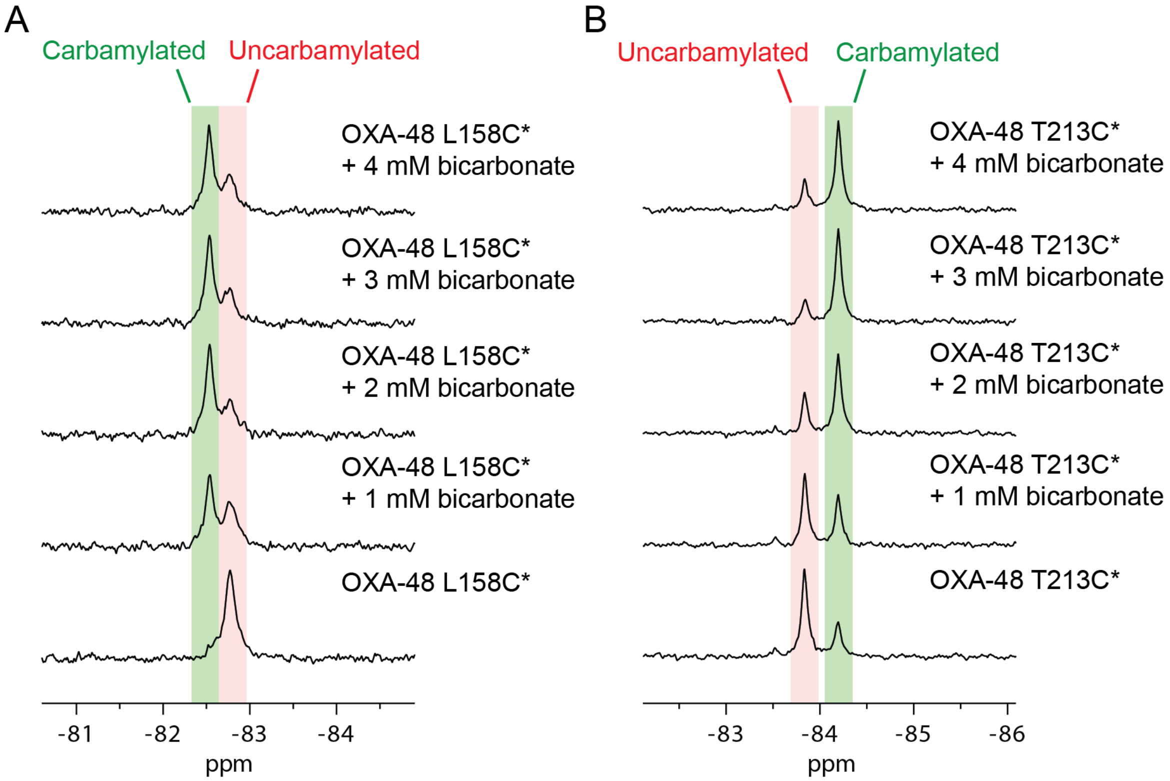

Figure S7. Impact of low pH on the carbamylation state of ^{19}F-labelled OXA-48 variants. ^{19}F-NMR spectra showing the impact of increasing concentrations of sodium bicarbonate on the carbamylation status of (A) OXA-48 L158C* and (B) OXA-48 T213C* in 50 mM sodium acetate, pH 4.5. In the absence of bicarbonate, the peak corresponding to carbamylated OXA-48 L158C* could not be clearly observed, while a clear peak was present in the corresponding spectrum of OXA-48 T213C*. Based on these NMR spectra, it appeared that carbamylation is relatively disfavoured with the OXA-48 L158C* variant, as compared to the T213C* variant.

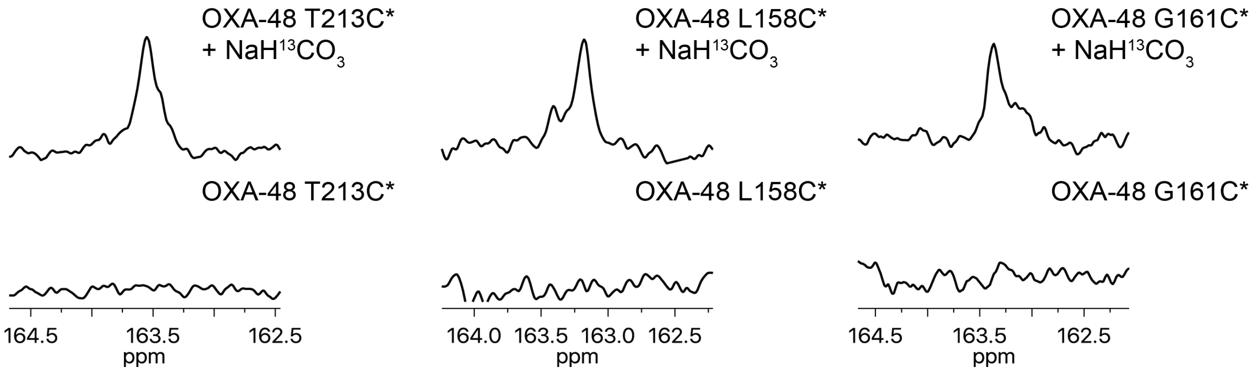

Figure S8. ^{13}C-NMR spectra of ^{19}F-labelled OXA-48 variants in ^{13}C-labelled sodium bicarbonate. Samples consisted of the indicated enzyme (OXA-48 T213C*: 760 µM; OXA-48 L158C*: 700 µM OXA-48 G161C*: 1.5 mM) and sodium bicarbonate (1 mM, if present) in 50 mM sodium phosphate, pH 7.5, 10 % D_2O. The ^{13}C signals observed following addition of $NaH^{13}CO_3$ are consistent with those previously observed for ^{13}C-carbamylated OXA-48.[12]

```
OXA-10      ---MKTF-----AAYVIIACLSSTA---------LAGSITENTSWNKEFSAEAVNGVFVL  43
OXA-23      MNKY--FTCYVVASLFLSGCTVQHNLINETP---SQIVQGHNQVIHQYFDEKNTSGVLVI  55
OXA-24/40   MKKFILPIFSISILVSLSACSSIKTKSEDNF---HISSQQHEKAIKSYFDEAQTQGVIII  57
OXA-48      MRVLALS-----AVFLVASIIGMPA---------VAKEWQENKSWNAHFTEHKSQGVVVL  46
OXA-51      --MNIKTLLLITSAIFISACSPYIVTANPNH--SASKSDEKAEKIKNLFNEVHTTGVLVI  56
OXA-58      -MKLLKILSLVCLSISIGACAEHSMSRAKTSTIPQVNNSIIDQNVQALFNEISADAVFVT  59
                            : .                        :  *      .*.:

OXA-10      CKSSSKSCATNDLARASKEYLPASTFKIPNAIIGLETGVIKNEHQVFKWDGKPRAMKQWE  103
OXA-23      QTDKKINLYGNALSRANTEYVPASTFKMLNALIGLENQK-TDINEIFKWKGEKRSFTAWE  114
OXA-24/40   KEGKNLSTYGNALARANKEYVPASTFKMLNALIGLENHK-ATTNEIFKWDGKKRTYPMWE  116
OXA-48      WNENKQQGFTNNLKRANQAFLPASTFKIPNSLIALDLGVVKDEHQVFKWDGQTRDIATWN  106
OXA-51      QQGQTQQSYGNDLARASTEYVPASTFKMLNALIGLEHHK-ATTTEVFKWDGQKRLFPEWE  115
OXA-58      YDGQNIKKYGTHLDRAKTAYIPASTFKIANALIGLENHK-ATSTEIFKWDGKPRFFKAWD  118
               .. .   . * **.  ::******: *::*.*:        ::***.*: *    *:

OXA-10      RDLTLRGAIQVSAVPVFQQIAREVGEVRMQKYLKKFSYGNQNISGGIDKFWLEGQLRISA  163
OXA-23      KDMTLGEAMKLSAVPVYQELARRIGLDLMQKEVKRIGFGNAEIGQQVDNFWLVGPLKVTP  174
OXA-24/40   KDMTLGEAMALSAVPVYQELARRTGLELMQKEVKRVNFGNTNIGTQVDNFWLVGPLKITP  176
OXA-48      RDHNLITAMKYSVVPVYQEFARQIGEARMSKMLHAFDYGNEDISGNVDSFWLDGGIRISA  166
OXA-51      KDMTLGDAMKASAIPVYQDLARRIGLELMSKEVKRVGYGNADIGTQVDNFWLVGPLKITP  175
OXA-58      KDFTLGEAMQASTVPVYQELARRIGPSLMQSELQRIGYGNMQIGTEVDQFWLKGPLTITP  178
            :* .*  *:  *.:**:*::**. *   *.. :: ..:** :*.  :*.*** * : ::
                                                β5 strand       β6 strand
OXA-10      VNQVEFLESLYLNKLSASKENQLIVKEALVTEAAPEYLVHSKTGFSGVGTESNPGVAWWV  223
OXA-23      IQEVEFVSQLAHTQLPFSEKVQANVKNMLLLEESNGYKIFGKTGWA---MDIKPQVGWLT  231
OXA-24/40   VQEVNFADDLAHNRLPFKLETQEEVKKMLLIKEVNGSKIYAKSGWG---MGVTPQVGWLT  233
OXA-48      TEQISFLRKLYHNKLHVSERSQRIVKQAMLTEANGDYIIRAKTGYS---TRIEPKIGWWV  223
OXA-51      QQEAQFAYKLANKTLPFSPKVQDEVQSMLFIEEKNGNKIYAKSGWG---WDVDPQVGWLT  232
OXA-58      IQEVKFVYDLAQGQLPFKPEVQQQVKEMLYVERRGENRLYAKSGWG---MAVDPQVGWYV  235
             :: .*  .*    *   . . *  *:. :  :      : .*:*:.       *  :.* .

OXA-10      GWVEKET-EVYFFAFNMDIDNESKLPLRKSIPTKIMESEGIIGG-       266
OXA-23      GWVEQPDGKIVAFALNMEMRSEMPASIRNELLMKSLKQLNII---       273
OXA-24/40   GWVEQANGKKIPFSLNLEMKEGMSGSIRNEITYKSLENLGII---       275
OXA-48      GWVELDD-NVWFFAMNMDMPTSDGLGLRQAITKEVLKQEKIIP--       265
OXA-51      GWVVQPQGNIVAFSLNLEMKKGIPSSVRKEITYKSLEQLGIL---       274
OXA-58      GFVEKADGQVVAFALNMQMKAGDDIALRKQLSLDVLDKLGVFHYL       280
            *:*     :   *::*:::       :*: :  . :..  ::
```

Figure S9. Sequence alignment of OXA-48 with other representative class D SBLs. The position of Thr213, which was substituted in the OXA-48 T213C* variant, is highlighted with a green background. Thr213 is positioned on the β5-β6 loop (Figure 1C, S1); residues of the β5 and β6 strands are shown with a blue background. The high level of sequence variation in the β5-β6 loop between different enzymes suggests this is an appropriate position for the ^{19}F-label. The nucleophilic serine and carbamylated lysine residues are shown with yellow backgrounds. Sequence alignment was performed using Clustal Omega (https://www.ebi.ac.uk/Tools/msa/clustalo/), and the β-lactamases sequences were retrieved from the Beta-Lactamase DataBase (BLDB).[13]

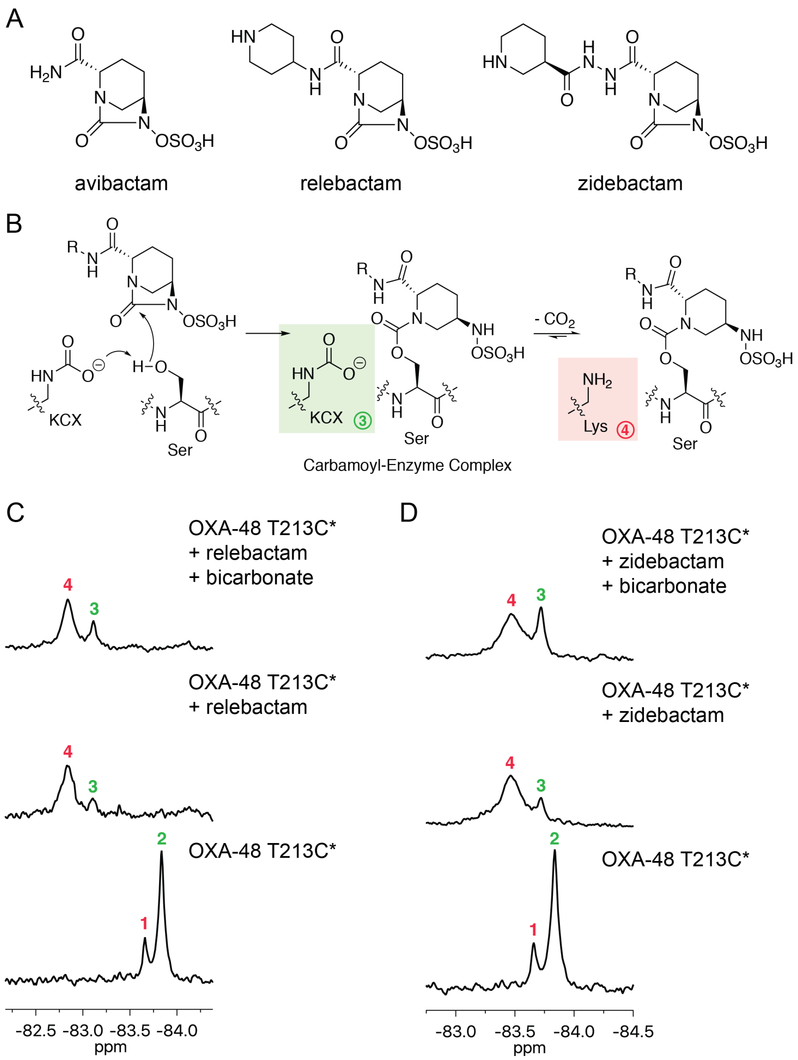

Figure S10. Interaction of OXA-48 T213C* with diazabicyclooctane inhibitors. (A) Chemical structures of avibactam and the related diazabicyclooctanes relebactam and zidebactam. (B) Scheme showing the interaction of diazabicyclooctanes with class D SBLs. The carbamylated and uncarbamylated states of the carbamoyl-enzyme complex are shown in green and red, respectively. ^{19}F-NMR spectra showing the impact of (C) relebactam and (D) zidebactam on OXA-48 T213C*, with and without added sodium bicarbonate. The signals arising from the complex derived from relebactam/zidebactam and OXA-48 T213C* were assigned as the carbamylated (3) and uncarbamylated (4) states. The assignments for OXA-48 T213C* in the absence of inhibitor are based on the spectra shown in Figure 1D.

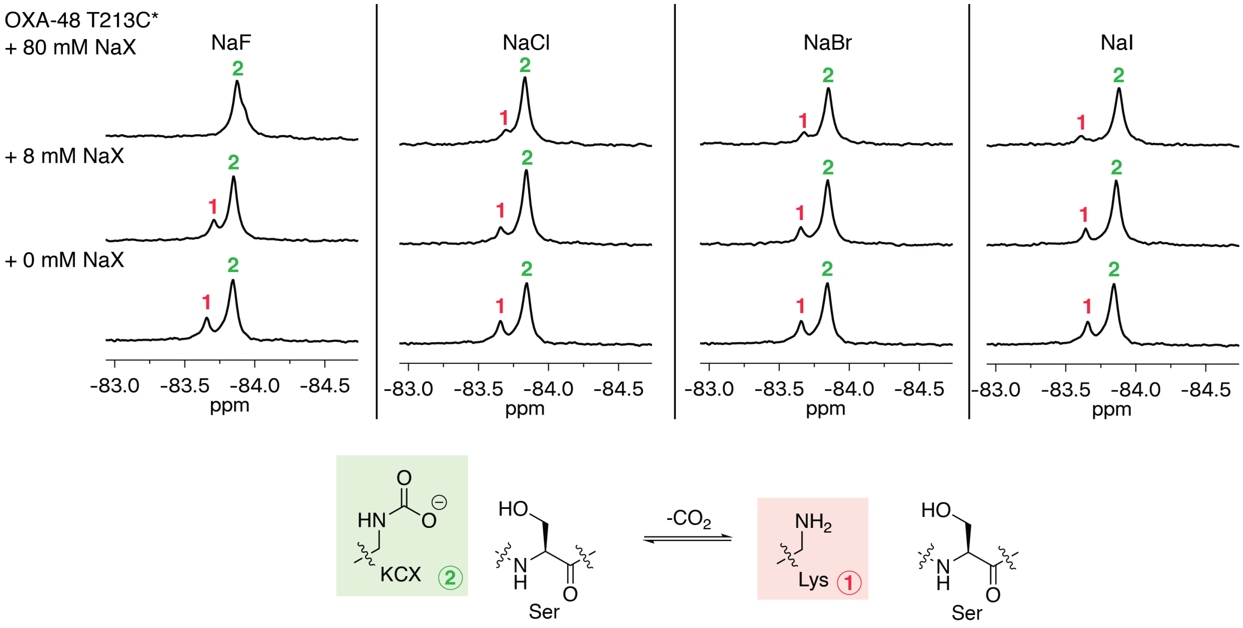

Figure S11. Impact of halide ions on the ^{19}F-NMR spectrum of OXA-48 T213C*. ^{19}F-NMR spectra showing the impact of sodium halides (8 mM or 80 mM) on OXA-48 T213C* (160 µM). Chloride, bromide, and iodide salts did not have a major impact on the ^{19}F chemical shifts of OXA-48 T213C*. The apparent impact of sodium fluoride may result from changes in pH associated with higher fluoride concentrations.

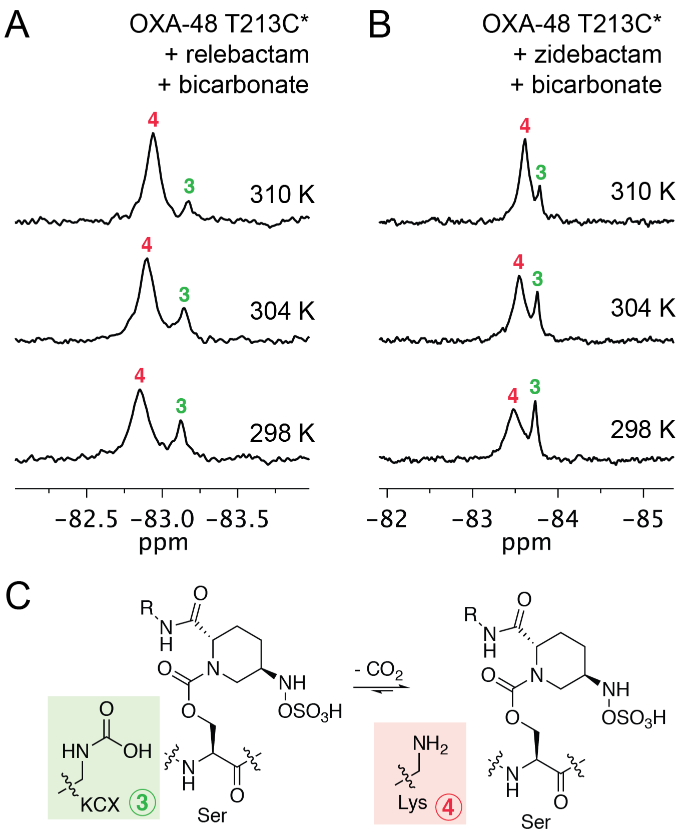

Figure S12. Impact of temperature on carbamylation of OXA-48 T213C* with relebactam and zidebactam. ^{19}F-NMR spectra showing the impact of temperature on the extent of carbamylation of OXA-48 T213C* (160 µM) in the presence of 800 µM (A) relebactam and (B) zidebactam, with 1 mM sodium bicarbonate. As the temperature of the sample was increased, the extent of carbamylation was observed to decrease (i.e., peak 3 decreased in intensity). (C) Proposed structures corresponding to the ^{19}F signals observed for OXA-48 T213C* with relebactam and zidebactam. These assignments are based in part on those shown in Figure S10. The R groups correspond to the structures of relebactam and zidebactam shown in Figure S10.

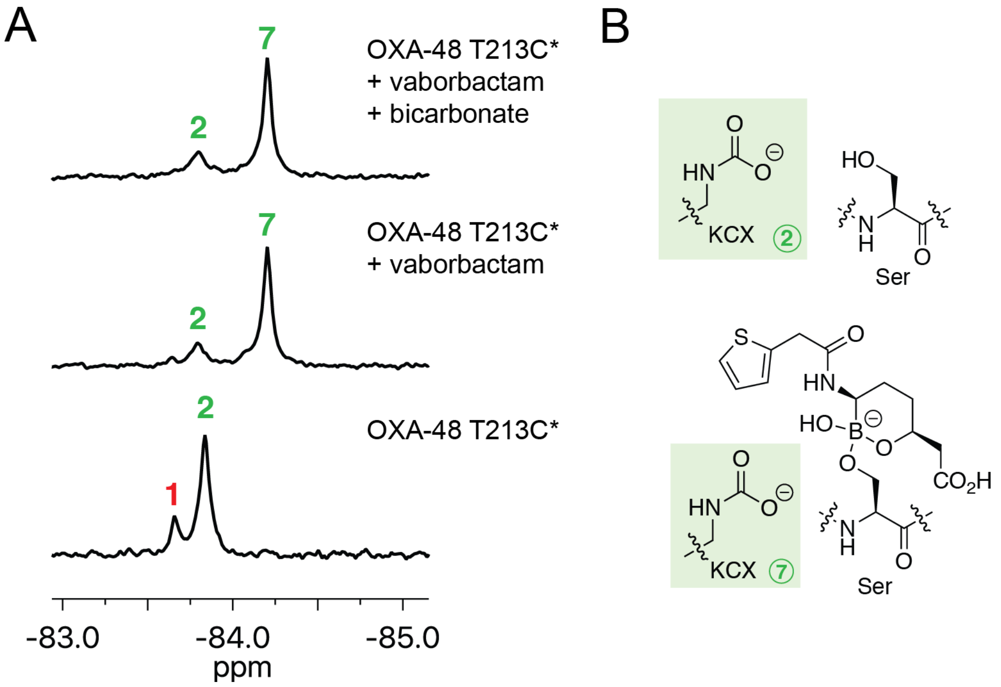

Figure S13. Impact of bicarbonate on the vaborbactam OXA-48 T213C* complex. (A) ^{19}F-NMR spectra showing OXA-48 T213C* (160 µM) in the presence of vaborbactam (1.12 mM), with and without 1 mM bicarbonate. Addition of bicarbonate was not observed to impact on the ^{19}F signals in the spectrum. (B) Proposed structures assigned to the ^{19}F signals observed by NMR. The proposed structure corresponding to signal 1 is shown in Figure 1D.

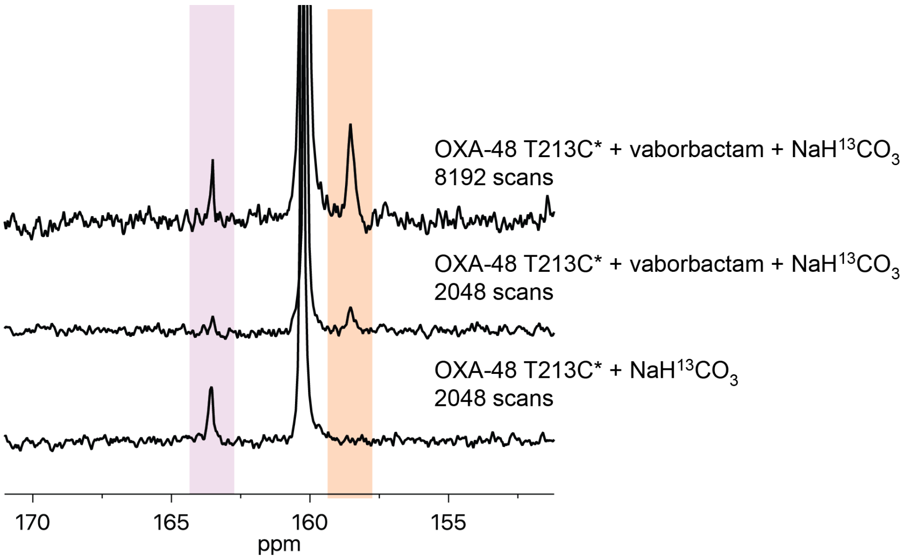

Figure S14. ^{13}C-NMR spectra of OXA-48 T213C* with vaborbactam. Samples consisted of 750 µM OXA-48 T213C*, 10 mM $NaH^{13}CO_3$, and 5 mM vaborbactam (if present; 100 mM stock prepared in DMSO-d_6), in 50 mM sodium phosphate, pH 7.5, 10 % D_2O. The peaks highlighted in purple are assigned as corresponding to carbamylated OXA-48 T213C* (as shown in Figure S8, and as described previously);[12] those in orange are assigned as corresponding to the complex of vaborbactam covalently bound to carbamylated OXA-48 T213C* (resembling what was observed previously for the carbamylated complex of a cyclic boronate with OXA-10).[14] The spectra acquired suggest that OXA-48 T213C* is not fully complexed with vaborbactam (due to the apparent presence of two peaks, and the relatively weak signal intensities), consistent with the relatively poor binding interaction shown in Figure S15. However, we cannot exclude the possibility that the binding of vaborbactam may impact the dynamics of OXA-48 T213C*, thereby impacting signal intensity and broadness.

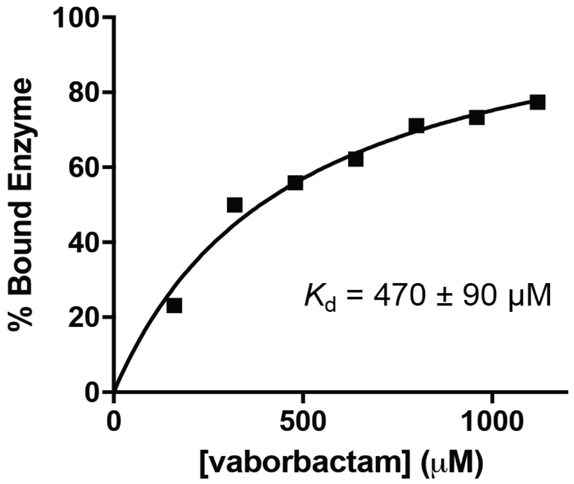

Figure S15. Binding curve for vaborbactam with OXA-48 T213C*. The extent of binding of vaborbactam to OXA-48 T213C* was determined by ^{19}F-NMR, based on the relative integration of the signals corresponding to the bound and unbound forms of the enzyme. The vaborbactam concentrations are as indicated on the chart, and 160 μM OXA-48 T213C* was used. Non-linear regression analysis was performed using Prism 7 (GraphPad), using the "One Site – Specific Binding" function.

Table S2. Processing and refinement statistics for OXA-48 T213C* crystallography.

Data Set	6RJ7
**Resolution (outer shell) (Å)**	66.41-1.73 (1.729 – 1.73)
**Unit cell dimensions**	64.36, 58.03, 66.43<br>90.0, 91.49, 90.0
**Space group**	$P\ 1\ 2_1\ 1$
**Protein molecules per ASU†**	2
**Completeness (outer shell) (%)**	99.74 (99.73)
**No. of unique reflections (outer shell)**	51207 (5108)
**Multiplicity (outer shell)**	16.80 (17.59)
R_{merge}	0.14
**I/σ mean (outer shell)**	16.25 (1.44)
**Wilson B**	22.61
**Refinement**	
**B factors:**	
**Overall**	28.75
**Protein**	28.25
**Ligand**	35.98
**Water**	33.59
**RMSD from ideal bond length (Å) ‡**	0.008
**RMSD from ideal angles (degrees)**	0.89
R_{work} **(%)**	17.74
R_{free} **(%)**	20.96

†ASU = asymmetric unit. ‡ RMSD = root mean square deviation.

References

[1] C. T. Lohans, H. T. H. Chan, T. R. Malla, K. Kumar, J. J. A. G. Kamps, D. J. B. McArdle, E. van Groesen, M. de Munnik, C. L. Tooke, J. Spencer, R. S. Paton, J. Brem, C. J. Schofield, *Angew. Chem. Int. Ed. Engl.* **2019**, *131*, 2012.

[2] J. Brem, W. B. Struwe, A. M. Rydzik, H. Tarhonskaya, I. Pfeffer, E. Flashman, S. S. van Berkel, J. Spencer, T. D. W. Claridge, M. A. McDonough, J. L. P. Benesch, C. J. Schofield, *Chem. Sci.* **2015**, *6*, 956.

[3] A. Makena, J. Brem, I. Pfeffer, R. E. Geffen, S. E. Wilkins, H. Tarhonskaya, E. Flashman, L. M. Phee, D. W. Wareham, C. J. Schofield, *J Antimicrob Chemother* **2015**, *70*, 463.

[4] G. M. Sheldrick, *Acta Cryst.* **2008**, *A64*, 112.

[5] A. J. McCoy, R. W. Grosse-Kunstleve, P. D. Adams, M. D. Winn, L. C. Storoni, R. J. Read, *J. Appl. Crystallogr.* **2007**, *40*, 658.

[6] D. T. King, A. M. King, S. M. Lal, G. D. Wright, N. C. J. Strynadka, *ACS Infect. Dis.* **2015**, *1*, 175.

[7] P. Emsley, B. Lohkamp, W. G. Scott, K. Cowtan, *Acta Crystallogr. D Biol. Crystallogr.* **2010**, *66*, 486.

[8] P. D. Adams, P. V. Afonine, G. Bunkóczi, V. B. Chen, I. W. Davis, N. Echols, J. J. Headd, L. W. Hung, G. J. Kapral, R. W. Grosse-Kunstleve, A. J. McCoy, N. W. Moriarty, R. Oeffner, R. J. Read, D. C. Richardson, J. S. Richardson, T. C. Terwilliger, P. H. Zwart, *Acta Crystallogr. D Biol. Crystallogr.* **2010**, *66*, 213.

[9] J. D. Docquier, V. Calderone, F. De Luca, M. Benvenuti, F. Giuliani, L. Bellucci, A. Tafi, P. Nordmann, M. Botta, G. M. Rossolini, S. Mangani, *Chem Biol* **2009**, *16*, 540.

[10] H. Buschmann, E. Dutkiewicz, W. Knoche, *Ber. Bunsenges. Phys. Chem.* **1982**, *86*, 129.

[11] C. T. Lohans, E. van Groesen, K. Kumar, C. L. Tooke, J. Spencer, R. S. Paton, J. Brem, C. J. Schofield, *Angew. Chem. Int. Ed. Engl.* **2018**, *57*, 1282.

[12] C. T. Lohans, D. Y. Wang, C. Jorgensen, S. T. Cahill, I. J. Clifton, M. A. McDonough, H. P. Oswin, J. Spencer, C. Domene, T. D. W. Claridge, J. Brem, C. J. Schofield, *Org Biomol Chem* **2017**, *15*, 6024.

[13] T. Naas, S. Oueslati, R. A. Bonnin, M. L. Dabos, A. Zavala, L. Dortet, P. Retailleau, B. I. Iorga, *J Enzyme Inhib Med Chem* **2017**, *32*, 917.

[14] S. T. Cahill, R. Cain, D. Y. Wang, C. T. Lohans, D. W. Wareham, H. P. Oswin, J. Mohammed, J. Spencer, C. W. Fishwick, M. A. McDonough, C. J. Schofield, J. Brem, *Antimicrob Agents Chemother* **2017**, *61*,
